# Therapeutic PD-L1 antibodies are more effective than PD-1 antibodies in blocking PD-1/PD-L1 signaling

**DOI:** 10.1038/s41598-019-47910-1

**Published:** 2019-08-07

**Authors:** Annika De Sousa Linhares, Claire Battin, Sabrina Jutz, Judith Leitner, Christine Hafner, Joshua Tobias, Ursula Wiedermann, Michael Kundi, Gerhard J. Zlabinger, Katharina Grabmeier-Pfistershammer, Peter Steinberger

**Affiliations:** 1 Division of Immune Receptors and T Cell Activation, Center for Pathophysiology, Infectiology, Medical University of Vienna, Vienna, Austria; 2 Division of Clinical and Experimental Immunology, Center for Pathophysiology, Infectiology, and Immunology, Institute of Immunology, Medical University of Vienna, Vienna, Austria; 3 Department of Dermatology, University Hospital St. Pölten, Karl Landsteiner University of Health Sciences, St. Pölten, Austria; 4 Karl Landsteiner Institute of Dermatological Research, Karl Landsteiner Gesellschaft, St. Pölten, Austria; 5 Institute of Specific Prophylaxis and Tropical Medicine, Center for Pathophysiology, Infectiology, and Immunology, Medical University of Vienna, Vienna, Austria; 6 Institute of Environmental Health, Center for Public Health, Medical University of Vienna, Vienna, Austria

**Keywords:** Immunotherapy, Tumour immunology

## Abstract

Inhibitors of PD-1 signaling have revolutionized cancer therapy. PD-1 and PD-L1 antibodies have been approved for the treatment of cancer. To date, therapeutic PD-1 inhibitors have not been compared in a functional assay. We used an efficient T cell reporter platform to evaluate the efficacy of five clinically used PD-1 inhibitors to block PD-1 signaling. The half maximal effective concentrations (EC_50_) for nivolumab and pembrolizumab were 76.17 ng/ml (95% CI 64.95–89.34 ng/ml) and 39.90 ng/ml (34.01–46.80 ng/ml), respectively. The EC_50_ values of the PD-L1 inhibitors were 6.46 ng/ml (5.48–7.61 ng/ml), 6.15 ng/ml (5.24–7.21 ng/ml) and 7.64 ng/ml (6.52–8.96 ng/ml) for atezolizumab, avelumab, and durvalumab, respectively. In conclusion, a functional assay evaluating antibodies targeting PD-1 inhibition *in vitro* revealed that pembrolizumab is a slightly more effective PD-1 blocker than nivolumab, and that PD-L1 antibodies are superior to PD-1 antibodies in reverting PD-1 signaling.

## Introduction

Programmed cell death-1 (PD-1), an important inhibitory receptor, is critical for the maintenance of central and peripheral T cell tolerance. However, PD-1, which is upregulated on activated and exhausted T cells, also limits productive immune responses against pathogens and cancer cells. PD-1 signaling is induced upon binding of its ligands, PD-1 ligand-1 (PD-L1) and PD-1 ligand-2 (PD-L2). The expression of PD-L2 is mainly restricted to professional antigen presenting cells (APCs) like macrophages and dendritic cells (DCs), whereas PD-L1 is also expressed in non-hematopoietic tissues and can thus be regarded as the major PD-1 ligand. Importantly, PD-L1 is also upregulated in the tumor microenvironment and is found in a large variety of tumor cells. Tumor infiltrating lymphocytes frequently express PD-1, providing a rationale for therapeutically disrupting the PD-1/PD-L1 interaction to improve anti-tumor responses. Currently, several PD-1 and PD-L1 antibodies are in clinical use for the treatment of various solid cancers and lymphomas, and blocking of the PD-1 pathway was shown to induce impressive response rates across a broad spectrum of tumor types^1,2^.

Although the clinical indications for which regulatory authorities have approved antibodies targeting PD-1 and PD-L1 only partially overlap, the mode of action of these drugs is blocking PD-1 signaling. Therefore, it is evident that the ability of these agents to disrupt the function of PD-1 critically impacts their clinical efficacy. As with other drugs, dose response curves and half maximal effective concentrations (EC_50_) provide valuable information on the efficacy of these antibodies. However, these values are not available for therapeutic PD-1 and PD-L1 antibodies, and the ability of these agents to block PD-1 signaling have not been compared in a functional assay. We have previously developed and described an efficient, robust and cost-effective fluorescent T cell reporter platform for the evaluation of immune checkpoint inhibitors (ICIs) targeting different inhibitory receptors, including PD-1^3^.

Here, we have exploited this system to determine the EC_50_ values of five antibody-drugs used in the clinic to block the PD-1/PD-L1 interaction, namely atezolizumab, avelumab, durvalumab, nivolumab and pembrolizumab. In addition, we used flow cytometry-based binding assays on the cells in our reporter system to explore whether it can be used to predict the functional potency of blocking antibodies.

## Results

### Characterization of PD-1 reporter cells and T cell stimulator cells expressing PD-1 ligands

**Figure 1:**
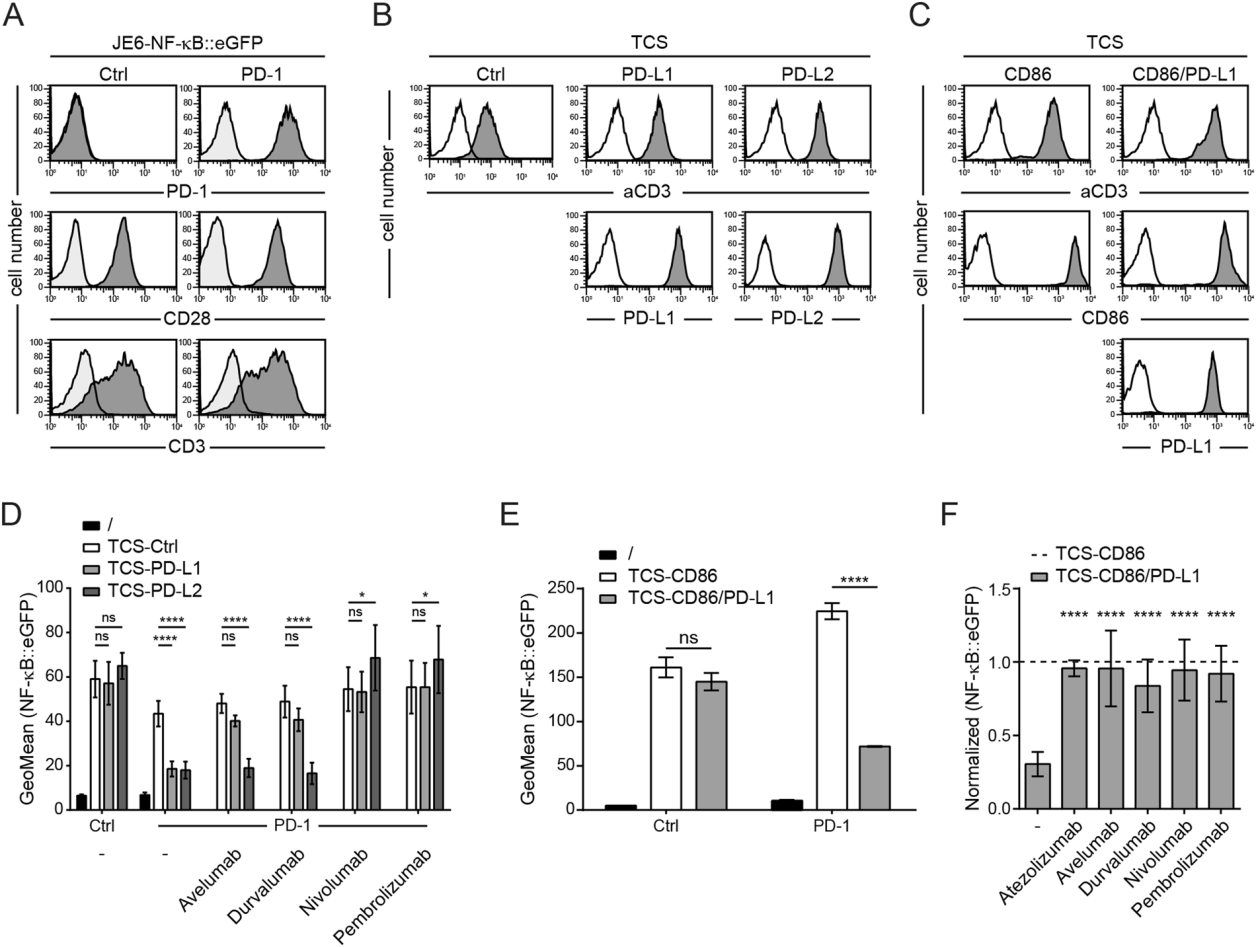
A T cell reporter platform to evaluate therapeutic PD-1 and PD-L1 antibodies. (**A**) Surface expression of PD-1, CD28 or CD3 on the control and PD-1 reporter cells is shown (dark grey histograms). Light grey histograms represent reactivity of isotype control antibodies. (**B**) Flow cytometric analysis of control TCS and TCS expressing PD-L1 or PD-L2. (**C**) Flow cytometric analysis of TCS-CD86 and TCS co-expressing CD86 and PD-L1. (**B**,**C**) Filled histograms: reactivity of antibodies to the indicated molecules. Open histograms: staining of control cells. (**D**) Control and PD-1 reporter cells were left unstimulated or stimulated with control TCS or TCS expressing PD-1 ligands (TCS-PD-L1 and TCS-PD-L2). PD-1 reporter cells were also stimulated in the presence of therapeutic antibodies to PD-1 and PD-L1 (used at 10 μg/ml). Results are shown for two independent experiments performed in triplicate. (**E**) Control and PD-1 reporter cells were stimulated with TCS expressing CD86 (TCS-CD86) and TCS co-expressing CD86 and PD-L1 (TCS-CD86/PD-L1). (**F**) PD-1 reporter cells were stimulated in absence and presence of the indicated PD-1 and PD-L1 antibodies (used at 10 μg/ml). Reporter gene expression upon stimulation with TCS-CD86/PD-L1 is shown normalized to reporter activation upon stimulation with TCS-CD86. Results shown in E and F are summarized from three independent experiments performed in duplicate. For statistical analysis an ordinary two-way ANOVA followed by Tukey’s multiple comparison test (**D**,**E**) and a one-way ANOVA followed by the Dunnett multiple comparison test (**F**) was used.

PD-1 expressing NF-κB::eGFP reporters and control reporters based on the human Jurkat T cell line JE6.1 were used in this study^3^. High levels of PD-1 were detected on the PD-1 reporters, whereas the control reporters were PD-1-negative. Both, the PD-1 and control reporters, expressed similar levels of the costimulatory receptor CD28 and of CD3 on their surface (Fig. 1A). To stimulate our reporters in the absence and presence of PD-1 ligands, we used T cell stimulators (TCS) based on the murine thymoma cell line BW5147^4^. TCS express a membrane-bound anti-human CD3 single chain antibody fragment that can functionally engage the CD3-TCR complex on our T cell reporters. TCS, which express high levels of human PD-L1 or PD-L2, were generated to trigger PD-1 on the PD-1 expressing reporter cells (Fig. 1B). In addition, we generated TCS expressing the costimulatory ligand CD86 and TCS co-expressing CD86 and PD-L1 (Fig. 1C).

To functionally validate our reporter platform, control and PD-1 reporter cells were stimulated by TCS expressing PD-1 ligands or control TCS. In the control reporter cells similar levels of reporter activation were induced by control TCS and TCS expressing PD-1 ligands. In contrast, eGFP expression on the PD-1 reporters was strongly reduced upon stimulation with TCS-PD-L1 or TCS-PD-L2 (Fig. 1D). This effect was completely abolished upon the addition of the therapeutic PD-1 antibodies nivolumab or pembrolizumab, whereas the therapeutic PD-L1 antibodies avelumab and durvalumab blocked PD-L1 but not PD-L2 inhibition (Fig. 1D). In a next set of experiments, we analyzed the effects of PD-1 on reporter cells that received costimulatory signals. For this we used TCS co-expressing PD-L1 and CD86, a ligand for the primary costimulatory receptor CD28. As expected, stimulation with TCS-CD86 resulted in strong activation of both reporters, and the presence of PD-L1 on these cells inhibited eGFP expression by the PD-1 reporters (Fig. 1E). This reduction in reporter gene expression was abolished by the presence of therapeutic PD-1 and PD-L1 antibodies (Fig. 1F). Taken together, our results indicate that PD-1 strongly suppressed reporter gene expression in the presence or absence of CD28 costimulation. Moreover, these experiments demonstrate that our reporter platform is an intrinsically controlled system well-suited to evaluate and compare clinically used antibodies targeting PD-1 inhibition.

### Determination of functional EC_50_ values for atezolizumab, avelumab, durvalumab, nivolumab and pembrolizumab

**Figure 2:**
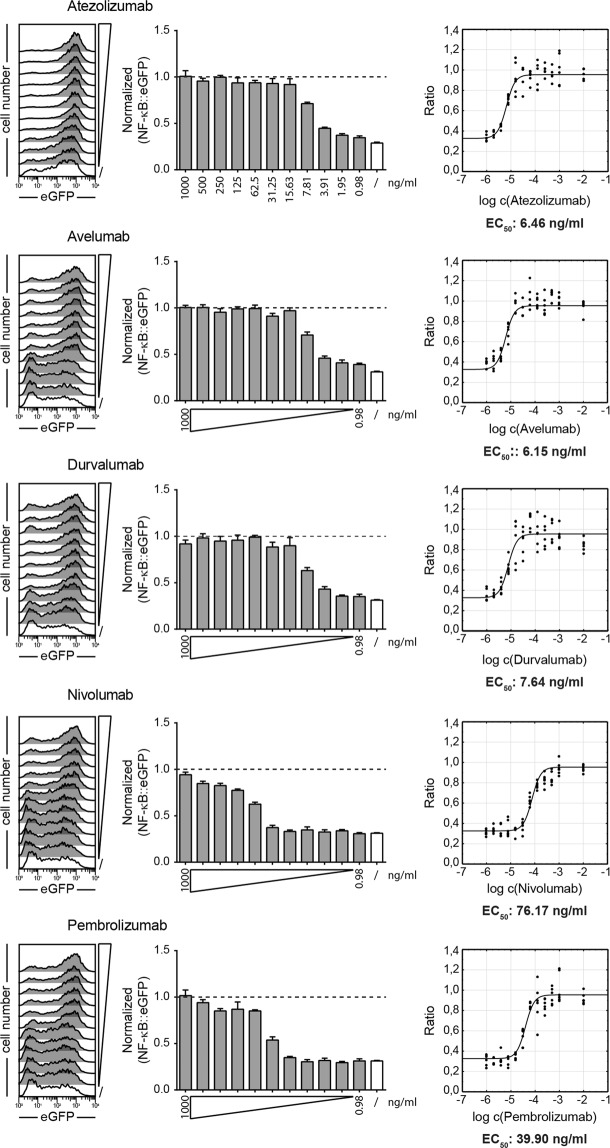
Determination of functional EC_50_ values for atezolizumab, avelumab, durvalumab, nivolumab and pembrolizumab. PD-1 expressing reporter cells were stimulated for 24 h with TCS-CD86 and TCS-CD86/PD-L1 in presence of the indicated PD-1 and PD-L1 blocking antibodies at final concentrations ranging from 1000 to 0.98 ng/ml. Left: Flow cytometric measurement of eGFP expression on PD-1 reporter cells stimulated with TCS-CD86/PD-L1 in the absence (/) or presence of PD-1 blockers used at different concentrations. Data shown are representative of three independent experiments performed in duplicate. Middle: eGFP expression of PD-1 reporter cells stimulated with TCS-CD86/PD-L1 in presence of the indicated concentrations of PD-1 inhibitors. Data were normalized to the eGFP expression of PD-1 reporter cells stimulated in absence of PD-L1 (stimulation with TCS-CD86; dotted line). Right: Inhibition curves and half maximum effective concentrations (EC_50_) were calculated for the PD-1 and PD-L1 antibodies from normalized data using a 4-parameter logistic function. Results shown in the middle and right panels represent summarized data from three independent experiments performed in duplicate.

In order to assess the functional efficacy of ICIs in blocking PD-1 signaling, we set out to determine the EC_50_ values for each of these antibodies using our reporter system. Two recent studies suggest that CD28 signaling is the preferred target of PD-1 inhibition^5,6^. Consequently, we have used TCS-CD86/PD-L1 to stimulate PD-1 reporter cells. Nivolumab, pembrolizumab, atezolizumab, avelumab, and durvalumab were added at concentrations ranging from 1 µg/ml to 976 pg/ml, and reporter gene expression was measured after 24 h by flow cytometry. An increase in eGFP expression was observed in the PD-1 reporter cells in the presence of PD-1 or PD-L1 antibodies in a dose-dependent manner and PD-1 inhibition was completely abolished at higher concentrations of antibodies (Fig. 2). Normalized reporter gene expression values from three independently performed experiments were combined and used to calculate the EC_50_ values for each antibody. The EC_50_ values calculated for all three PD-L1 antibodies were very low at 6.46 ng/ml for atezolizumab, 6.15 ng/ml for avelumab and 7.64 ng/ml for durvalumab. The PD-1 antibodies were less effective with nivolumab having the highest EC_50_ value (76.17 ng/ml), while pembrolizumab (39.90 ng/ml) was slightly more effective (Fig. 2). In additional experiments, we evaluated avelumab and nivolumab using different T cell stimulator cells based on the human myelogenous leukemia cell line K562. The EC_50_ values obtained from these experiments were similar to those obtained with the TCS based on the BW5147 murine cell line (Fig. S1).

### Evaluation of therapeutic PD-1 and PD-L1 antibodies in a flow cytometry-based binding assay

**Figure 3:**
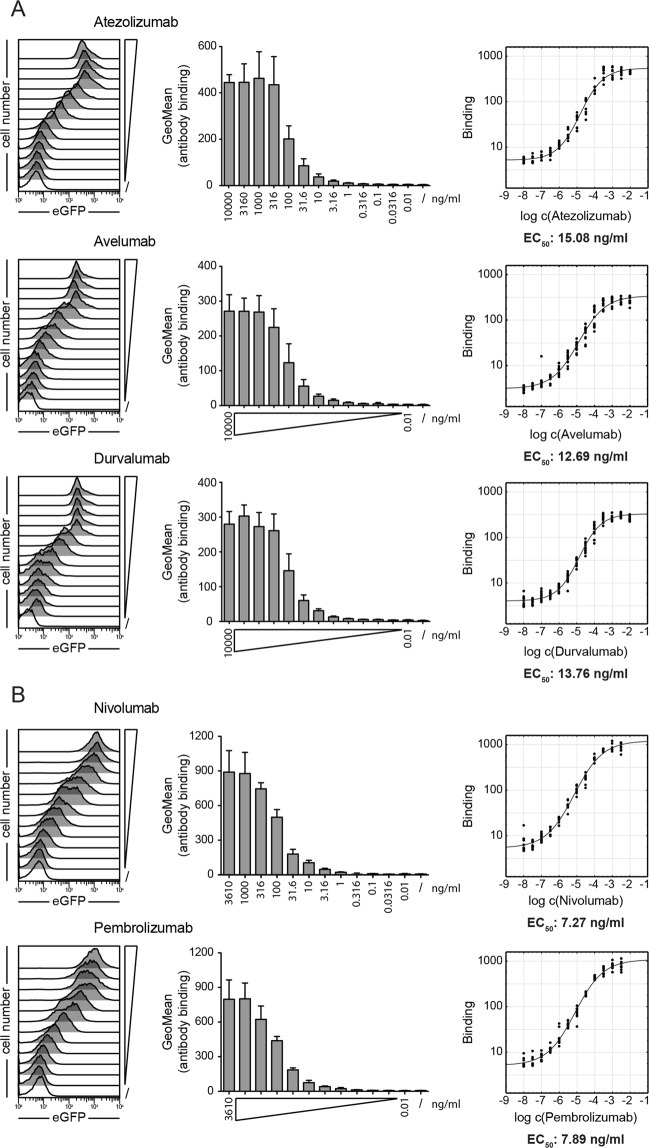
Binding of therapeutic PD-1 and PD-L1 antibodies to their targets. (**A**) TCS-PD-L1 were incubated with the indicated therapeutic PD-L1 antibodies used at final concentrations ranging from 10 μg/ml to 10 pg/ml. (**B**) PD-1 reporter cells were incubated with the indicated therapeutic PD-1 antibodies used at final concentrations ranging from 3.16 μg/ml to 10 pg/ml. (**A**,**B**) Left: histograms of the interaction of the indicated antibodies with TCS-PD-L1 or PD-1 reporters, respectively. Data shown are representative of three independent experiments performed in triplicate. Middle: bar diagrams show the gMFI values from three independent experiments performed in triplicate. Right: half maximum effective concentrations (EC_50_) were calculated from the binding data shown in the middle panels using a 4-parameter logistic function.

We hypothesized that the binding of blocking antibodies to their target antigens would correlate with their activity in our functional assay. To test this, we used reporter cells and T cell stimulator cells to perform binding assays with therapeutic antibodies targeting PD-1 and PD-L1, respectively. Atezolizumab, avelumab, and durvalumab binding was analyzed on PD-L1 expressing TCS, whereas PD-1 expressing reporter cells were used to test nivolumab and pembrolizumab binding. Target cells were probed with antibodies at a wide range of concentrations, and binding was detected with an APC-labeled anti-human IgG antibody and measured by flow cytometry. These experiments revealed comparable EC_50_ values for the PD-L1 antibodies atezolizumab (15.08 ng/ml), avelumab (12.69 ng/ml) and durvalumab (13.76 ng/ml) (Fig. 3A). The EC_50_ values for the PD-1 antibodies nivolumab (7.27 ng/ml) and prembrolizumab (7.89 ng/ml) were also similar (Fig. 3B).

### Comparison of EC_50_ values obtained in binding assays and functional assays

The EC_50_ values and the 95% CI from the functional assays and the binding assays are summarized in Table 1. A statistical comparison of these values is shown in Table 2. Our data show that among the therapeutic PD-1 antibodies, pembrolizumab is the most effective at blocking PD-1 signaling (Table 2). The functional EC_50_ values of the therapeutic PD-L1 antibodies were very similar and were significantly lower than the EC_50_ values of the therapeutic PD-1 antibodies (Tables 1 and 2). The results of the binding studies yielded similar EC_50_ values for the binding of both therapeutic PD-1 antibodies to PD-1 and for the binding of the three PD-L1 antibodies to PD-L1 (Table 1). The EC_50_ values of the PD-1 antibodies were significantly lower than the EC_50_ values of the PD-L1 antibodies (Table 2). Interestingly, neither the higher blocking ability of pembrolizumab compared to nivolumab nor the better performance of PD-L1 antibodies compared to PD-1 antibodies were reflected by the binding studies. This indicates that testing PD-1 blockers in standard flow cytometry-based binding assays may be of little use in appraising their ability to block PD-1 signaling in a functional assay.

**Table 1:** EC_50_ values for therapeutic PD-L1 and PD-1 antibodies estimated for their ability to block the PD-1/PD-L1 interaction in a functional assay and for their binding to their respective target antigens. Data from three independent experiments performed in duplicate were used to calculate EC_50_ values.

	Functional EC_50_ values		Binding EC_50_ values	
	EC_50_ (ng/ml)	95% CI	EC_50_ (ng/ml)	95% CI
Atezolizumab	6.46	5.48–7.61	15.08	12.55–18.12
Avelumab	6.15	5.24–7.21	12.69	10.35–15.56
Durvalumab	7.64	6.52–8.96	13.76	12.03–15.75
Nivolumab	76.17	64.95–89.34	7.27	5.94–8.90
Pembrolizumab	39.90	34.01–46.80	7.89	6.52–9.56

**Table 2:** EC_50_ values for functional blocking and for binding of therapeutic PD-1 and PD-L1 antibodies were compared using Walsh tests, and P-values for pairwise comparisons were Bonferroni-Holm corrected.

		Atezolizumab	Avelumab	Durvalumab	Nivolumab	Pembrolizumab
Function	**Atezolizumab**	—	0.674	0.147	<0.001	<0.001
	**Avelumab**	0.674	—	0.058	<0.001	<0.001
	**Durvalumab**	0.147	0.058	—	<0.001	<0.001
	**Nivolumab**	<0.001	<0.001	<0.001	—	<0.001
	**Pembrolizumab**	<0.001	<0.001	<0.001	<0.001	—
Binding	**Atezolizumab**	—	0.218	0.431	<0.001	<0.001
	**Avelumab**	0.218	—	0.516	<0.001	<0.001
	**Durvalumab**	0.431	0.516	—	<0.001	<0.001
	**Nivolumab**	<0.001	<0.001	<0.001	—	0.560
	**Pembrolizumab**	<0.001	<0.001	<0.001	0.560	—

## Discussion

T cell-expressed inhibitory receptors like CTLA-4, LAG-3, BTLA, TIM-3 and PD-1 function as immune checkpoints, which limit and terminate immune responses. We have recently highlighted that each of these molecules has unique properties and that many aspects of the roles of these molecules in immunity are currently not fully understood^7^. Immune checkpoint inhibition with therapeutic antibodies is a very effective mean to enhance T cell responses and it has been successfully exploited to treat patients suffering from different types of cancers^8–10^. Several antibodies targeting PD-1 or PD-L1 have received clinical approval as first and second line treatments for different malignancies and numerous clinical trials are ongoing to test the efficacy of these drugs when used alone or in combination with conventional anti-cancer drugs as well as targeted therapies^10^. Clinicians can choose from several ICIs to disrupt PD-1 signaling and a thorough characterization of these drugs is warranted to ensure their optimal use. Their efficacy in blocking the interaction of PD-1 with its major ligand PD-L1 is of great interest in this context. However, to our knowledge this information was not available for the PD-1 and PD-L1 antibodies that are currently used in the clinic.

We have previously developed a fluorescent transcriptional reporter system based on the human Jurkat T cell line^3,11,12^. When PD-1 is expressed in these cells, transcriptional activation is inhibited in the presence of PD-1 ligands. Analysis of PD-1 signaling in a multiparameter reporter line that simultaneously measures three transcription factors that play major roles in T cell activation, NF-κB, NFAT and AP-1, revealed that NF-κB and NFAT activation were both strongly inhibited by PD-1 engagement and are thus well-suited as a readout for PD-1 blocking^11^. In the current study, we used a highly sensitive NF-κB::eGFP JE6.1 reporter cell line expressing PD-1 to determine the EC_50_ values for the five antibodies that are currently used in the clinic to disrupt PD-1 signaling^3^. T cell stimulator cells expressing human PD-L1 and providing CD28 costimulation via CD86 were used to activate these reporters in presence of different concentrations of the ICIs. We found that the PD-1 antibody pembrolizumab is slightly more effective than nivolumab and that the PD-L1 antibodies atezolizumab, avelumab and durvalumab have significantly lower EC_50_ values than the PD-1 antibodies. Experiments with the PD-L1 antibody avelumab and the PD-1 antibody nivolumab using different T cell stimulator cells based on the human K562 cell line yielded similar EC_50_ values and confirmed that the PD-L1 antibody had a higher blocking efficiency than the PD-1 antibody (Fig. S1). K562 cells are devoid of CD80 or CD86 so these results also suggest that CD28 costimulation does not have a major influence on the functional EC_50_ values for PD-1 and PD-L1 antibodies. One potential explanation for the lower functional EC_50_ values of PD-L1 antibodies compared to PD-1 antibodies is that ligands are more effectively blocked than receptors, but more work is required to address this possibility. We hypothesized that the binding activity of the blocking antibodies to their target antigens would correlate with their activity in our functional assay. We thus used the reporter cells and T cell stimulator cells to perform flow cytometry-based binding assays with ICIs targeting PD-1 and PD-L1. Surprisingly these experiments did not indicate that flow cytometry-based binding assays performed under standard conditions are informative regarding the EC_50_ values of PD-1 blockers.

Therapeutic antibodies targeting PD-1 inhibition have not been compared in clinical trials. Although analysis of published data on non-small-cell lung carcinoma (NSLC) patients did not reveal differences in the efficacy between PD-1 and PD-L1 antibodies, such studies have many limitations as pointed out by the authors^13,14^. Our results demonstrate a superior blocking capacity of therapeutic PD-L1 antibodies, but the distinct properties and functions of ICIs binding PD-1 and PD-L1 have to be considered when evaluating their clinical use. In the priming phase, T cells interact with dendritic cells which express both PD-1 ligands, PD-L1 and PD-L2^15^. Although there is ample evidence that blocking PD-L1 enhances the response of human T cells interacting with APCs, PD-1 antibodies, which block PD-1 binding to both ligands, are likely to be more effective at promoting the priming of tumor specific T cells^15–18^. It should be stressed however, that PD-1 expression does not selectively mark dysfunctional cells as it is not limited to chronically stimulated T cells, which enter a state often referred to as “exhaustion”^19,20^. All T cells upregulate this immune checkpoint upon activation and therefore PD-1 prevents the activation of self-reactive or pathognomonic T cells^21–23^. Thus, blockade of both PD-1 ligands on DCs and other APCs, mediated by PD-1 antibodies, could potentially be associated with more severe side effects than the use of PD-L1 antibodies, which do not prevent the binding of PD-1 to PD-L2. In line with this, there is some evidence that PD-1 antibodies induce higher rates of immune adverse events like pneumonitis than PD-L1 antibodies^13,14^. In the tumor microenvironment and on tumor cells, PD-L1 is the predominant PD-1 ligand and thus blocking PD-L1 and PD-1 is likely to be equally efficient in the effector phase of tumor specific T cells. Therapeutic PD-1 and PD-L1 antibodies currently in use also have different isotypes. PD-1 antibodies are IgG4, whereas the PD-L1 antibodies harbor unmodified (avelumab) or modified IgG1 Fc sequences (durvalumab and atezolizumab).

In addition to PD-1, PD-L1 also binds CD80, a molecule which has an important role as a costimulatory ligand^24,25^. Therapeutic PD-L1 antibodies also disrupt the binding of PD-L1 to CD80, and therefore PD-L1 antibodies could potentially also augment T cell responses by blocking this pathway.

PD-L1 blockers are used at much higher doses than PD-1 antibodies, while the results of our study reveal that PD-L1 antibodies have a superior blocking capacity. The serum half-life of IgG1 and IgG4 antibodies is 21 days and the reported half-lives of nivolumab, pembrolizumab, atezolizumab, and durvalumab were in this range, while a much lower half-life was reported for avelumab (Table 3). It is possible that other factors like the higher density of PD-L1 compared to PD-1 or the faster clearance of PD-L1-IgG1 antibodies in the tissues via Fc-receptor mediated mechanisms warrant a higher dosage of PD-L1 antibodies compared to PD-1 antibodies. Nevertheless, the results of our study may provide a rationale for testing PD-L1 antibodies at lower doses.

**Table 3:** Characteristics, dosage and indications of therapeutic antibodies targeting PD-1 and PD-L1.

Target	Name	Isotype	Serum half life^a^	Indication	Dosage	Weekly dosage^b^
PD-1	Nivolumab (Opdivo®)	human IgG4κ	26.7 d	melanoma, NSCLC, renal cancer, Hodgkin lymphoma, head and neck cancer, urothelial carcinoma	3 mg/kg e.o.w.^c^	1.5 mg/kg
	Pembrolizumab (Keytruda®)	humanized IgG4κ	25.8 d	melanoma, NSCLC, Hodgkin lymphoma, urothelial carcinoma	2 mg/kg every 3 w.^d^	0.67 mg/kg
PD-L1	Atezolizumab (Tecentriq®)	humanized IgG1κ	27 d	urothelial carcinoma, NSCLC	1200 mg every 3 w.	5.0 mg/kg
	Avelumab (Bavencio®)	human IgG1λ	6 d	Merkel cell carcinoma	10 mg/kg e.o.w.	5.0 mg/kg
	Durvalumab (Imfinzi®)	human IgG1κ	17 d	urothelial carcinoma, NSCLC	10 mg/kg e.o.w.	5.0 mg/kg

^a^Based on data submitted to the FDA.

^b^Based on an 80 kg body weight.

^c^Recently approved fixed dose: 240 mg e.o.w or 480 mg every 4 weeks.

^d^Recently approved fixed dose: 200 mg every 3 weeks.

abbreviations: e.o.w., every other week; w., weeks; d, days.

We show here, that the ICIs that are currently used to target the PD-1/PD-L1 interaction are extremely efficient blockers of PD-1 inhibition. Cmax values for pembrolizumab measured in sera of treated patients were more than three orders of magnitudes higher than the EC_50_ values measured in our *in vitro* assays^26^. Based on studies in mice, the concentration of these antibodies in the tumor microenvironment can be expected to be much lower than the Cmax values^27^. Information on the concentrations of PD-1 or PD-L1 antibodies in the tumors of treated patients is not available, and thus it is not known whether they are indeed higher than the EC_50_ values obtained in our *in vitro* assays. Nevertheless, the results of our study may provide a rationale for testing antibodies targeting PD-1 inhibition and in particular PD-L1 antibodies at lower doses. Various factors, such as hypoxia or difficulty in tumor stroma penetration, restrict the immunotherapy effects and measures to overcome these limitations; for instance, using antibody drug conjugates or prodrugs based disruption of hypoxia were shown to have utility in improving immune checkpoint blockade^28,29^.

Currently, there are intense efforts to develop novel ICIs targeting inhibitory pathways like LAG-3, TIM-3, TIGIT, BTLA or VISTA^7,10,30,31^. Selecting ICIs that are highly effective at blocking these pathways will greatly increase the prospects of such endeavors. Cellular reporter platforms such as the one used here are well-suited to discern ICIs with high potential.

## Material and Methods

### Antibodies, cell culture and flow cytometry

The Jurkat E6.1 cells and the BW5417 cell line, which is a murine thymoma cell line (short designation in this work: BW) were derived from inhouse stocks and cultured as previously described^11,32^. The generation and validation of Jurkat E6.1 NF-κB::eGFP, and Jurkat E6.1 NF-κB::eGFP-PD1 reporter T cell lines have been previously reported^3^. T cell stimulator cells (TCS), which are BW cells engineered to stably express an anti-human CD3 single chain fragment have previously been described in detail^4^. TCS expressing high levels of PD-L1, PD-L2, CD86 and co-expressing PD-L1 and CD86 or no human costimulatory molecule (TCS-control) were generated by retroviral transduction. Surface expression was confirmed by flow cytometry. All cell lines were tested for the absence of mycoplasma using a method described recently^33^. The cells were stained with a panel of antibodies to authenticate them, and the reporter and stimulator cells were kept in culture for up to three months without perceptible loss of functionality. The following antibodies from Biolegend (San Diego, CA) were used: APC-conjugated PD-1 (#EH12.2H7), APC-conjugated PD-L1 (#29E.2A3), APC-conjugated CD86 (#IT2.2), PE-Cy7-conjugated CD3 (#UCHT-1), PE-conjugated PD-L2 (#24 F.10C12), PE-conjugated CD28 (#28.2), and the PE-conjugated isotype antibody control. A DyLight-649-conjugated goat-anti-mouse IgG (H + L) antibody (Jackson ImmunoResearch, West Grove, PA) was used to detect the membrane-bound anti-CD3 fragment. An APC-conjugated antibody to mouse CD45 (#104, Biolegend) was used to exclude TCS in reporter assays.

The PD-1 antibodies, nivolumab (Opdivo®, Bristol-Myers Squibb GmbH & Co) and pembrolizumab (Keytruda®, MSD Sharp & Dohme GmbH), and the PD-L1 antibodies, avelumab (Bavencio®, Merck), durvalumab (Imfinzi®, AstraZeneca), and atezolizumab (Tecentriq®, Roche) were used at the indicated final concentrations.

Flow cytometry was performed on a FACSCalibur flow cytometer (Becton Dickinson Immunocytometry System, San Jose, CA) using CellQuest software. Data were analyzed with FlowJo (version 10.0.6, Tree Star, Ashland, OR) and GraphPad Prism (version 5, GraphPad Software, Inc., La Jolla, CA).

### Reporter assays

Reporter cells (5 × 10^4^) and TCS (2 × 10^4^) were cocultured in 96-well flat bottom plates for 24 h in the presence or absence of antibodies to PD-1 (nivolumab; pembrolizumab) or PD-L1 (avelumab; durvalumab; atezolizumab) at 1000, 500, 250, 125, 62.5, 31.25, 15.63, 7.81, 3.91, 1.95, and 0.98 ng/ml (two-fold dilution steps). Subsequently, reporter gene expression (eGFP) was analyzed by flow cytometry as previously described in detail^3^. Briefly, cells were harvested and TCS were excluded using mCD45 mAb. The geometric mean of the fluorescence intensity (gMFI) of viable reporter cells was used for further analysis. To estimate EC_50_ values, three independent PD-1 reporter stimulation experiments were performed in duplicate. For each stimulation experiment, reporter gene induction in response to stimulation in presence of PD-L1 (stimulation with TCS-CD86/PD-L1) was normalized to reporter gene expression in the respective control (stimulation with TCS-CD86) and expressed as fold induction of the (gMFI).

### Binding assays

PD-1 expressing reporter cells (1 × 10^5^) were incubated for 30 minutes at 4 °C with the PD-1 antibodies nivolumab or pembrolizumab at final concentrations of 3.16 µg/ml, 1 µg/ml, 316 ng/ml, 100 ng/ml, 31.6 ng/ml, 10 ng/ml, 3.16 ng/ml, 1 ng/ml, 316 pg/ml, 100 pg/ml, 31.6 pg/ml, and 10 pg/ml. All the PD-L1 antibodies, avelumab, durvalumab, and atezolizumab were incubated with PD-L1 expressing TCS at final concentrations of 10 µg/ml, 3.16 µg/ml, 1 µg/ml, 316 ng/ml, 100 ng/ml, 31.6 ng/ml, 10 ng/ml, 3.16 ng/ml, 1 ng/ml, 316 pg/ml, 100 pg/ml, 31.6 pg/ml and 10 pg/ml. Binding was detected using an APC-conjugated goat-anti-human IgG (Fc-specific) antibody (Jackson ImmunoResearch) measured via flow cytometry. Three independent PD-1 and PD-L1 antibody binding experiments were performed in triplicate. The geometric mean of the fluorescence intensity of viable reporter cells was used for further analysis.

### Statistics

A 4-parameter logistic function was fitted to assess the relationship between log concentration (mg/ml) and binding (gMFI) or normalized stimulation. The fit was excellent with a pseudo-R² above 0.9. EC_50_ values were estimated from the intercept and slope of the logistic function and 95% confidence intervals were computed by applying Fieller’s theorem. EC_50_ values for different monoclonal Abs were statistically compared using Walsh tests. P-values for pairwise comparisons were Bonferroni-Holm corrected. Analyses were done using SPSS 25.0 (IBM, Amonk, N.Y.).

## Supplementary information

Dataset 1
