# Supplementary material for: Therapeutic PD-L1 antibodies are more effective than PD-1 antibodies in blocking PD-1/PD-L1 signaling
Source: Sci Rep. 2019 Aug 7;9:11472. doi: 10.1038/s41598-019-47910-1 (PMC6685986; doi:10.1038/s41598-019-47910-1)
Supplement: Supplementary file 1 — Dataset 1 [file 41598_2019_47910_MOESM1_ESM.pdf]

Therapeutic PD-L1 antibodies are more effective than PD-1 antibodies in blocking PD-1/PD-L1 signaling

Annika De Sousa Linhares[1§], Claire Battin[1§], Sabrina Jutz[1], Judith Leitner[1], Christine Hafner[3,4], Joshua Tobias[5], Ursula Wiedermann[5], Michael Kundi[6], Gerhard J. Zlabinger[2], Katharina Grabmeier-Pfistershammer[2] and Peter Steinberger[1]*.

[1]Division of Immune Receptors and T Cell Activation, Center for Pathophysiology, Infectiology, Medical University of Vienna, Vienna, Austria
[2]Division of Clinical and Experimental Immunology, Center for Pathophysiology, Infectiology, and Immunology, Institute of Immunology, Medical University of Vienna, Vienna, Austria
[3]Department of Dermatology, University Hospital St. Pölten, Karl Landsteiner University of Health Sciences, St. Pölten, Austria
[4]Karl Landsteiner Institute of Dermatological Research, Karl Landsteiner Gesellschaft, St. Pölten, Austria
[5]Institute of Specific Prophylaxis and Tropical Medicine, Center for Pathophysiology, Infectiology, and Immunology, Medical University of Vienna, Vienna, Austria
[6]Institute of Environmental Health, Center for Public Health, Medical University of Vienna, Vienna, Austria
[§]These authors contributed equally and share first authorship

*Address correspondence to Peter Steinberger, Division of Immune Receptors and T Cell Activation, Center for Pathophysiology, Infectiology, Medical University of Vienna, Lazarettgasse 19, 1090 Vienna, Austria. Phone ++43-1-40160-33241; E-mail: peter.steinberger@meduniwien.ac.at

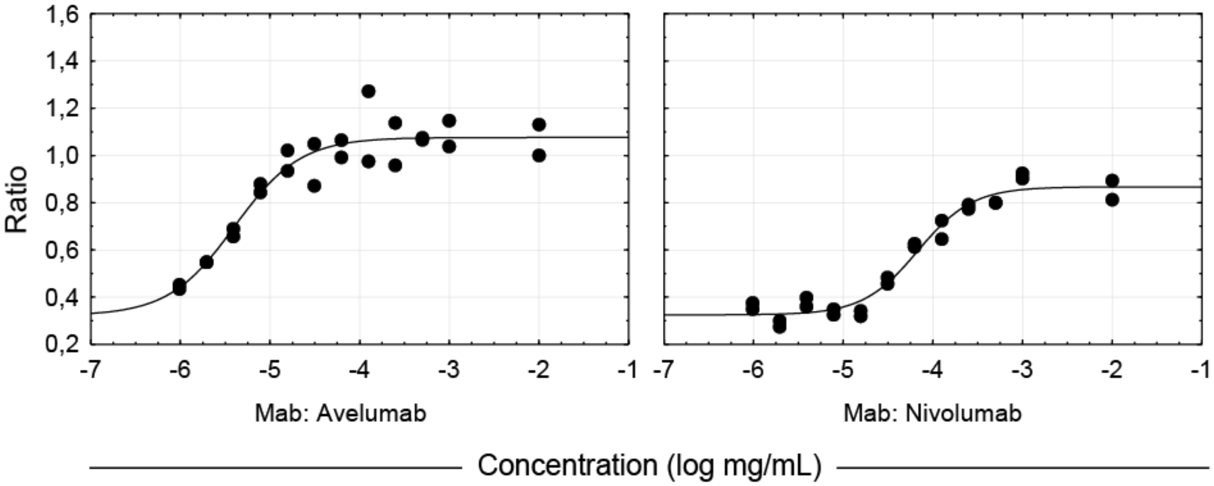

	EC50 (ng/ml)	95% CI
**Avelumab**	4.01	1.50-10.76
**Nivolumab**	67.92	51.47-89.64

	Avelumab	Nivolumab
**Avelumab**	-	<0.001
**Nivolumab**	<0.001	-

Supplemental Figure 1:
Determination of functional EC_{50} values for avelumab and nivolumab using T cell stimulator cells based on the human myelogenous leukemia cell line K562 (K-TCS). K-TCS were generated by expressing a membrane-bound anti-CD3 construct in K562 cells, which do not express CD80 or CD86. PD-1 expressing reporter cells were stimulated with K-TCS or K-TCS-PD-L1 for 24 hours in presence of PD-L1 antibody avelumab or PD-1 antibody nivolumab used in final concentrations ranging from 1000 to 0.98 ng/ml. Inhibition curves and half maximum effective concentrations (EC50) were calculated from normalized data and statistical analysis was performed as described in the material and methods section. Data are representative for three independently performed experiments.
